# Elevated GRP78 expression is associated with poor prognosis in patients with pancreatic cancer

**DOI:** 10.1038/srep16067

**Published:** 2015-11-04

**Authors:** Zheyu Niu, Mengyi Wang, Li Zhou, Lutian Yao, Quan Liao, Yupei Zhao

**Affiliations:** 1 Department of General Surgery, Peking Union Medical College Hospital, Chinese Academy of Medical Science & Peking Union Medical College, Beijing 100730, China

## Abstract

Glucose-regulated protein 78 (GRP78) is a member of the heat-shock protein 70 family. We evaluated the expression of GRP78 using tissue microarray-based immunohistochemistry in tumor tissues and adjacent nontumor tissues from 180 pancreatic ductal adenocarcinoma (PDAC) patients. The associations between the expression levels of GRP78, clinicopathological factors, and overall survival were evaluated. The results showed that the expression of GRP78 was significantly higher in PDAC cells than in normal pancreatic duct cells within adjacent nontumor tissues (p < 0.05). The increased expression of GRP78 in the tumor tissues was significantly correlated with a higher T-stage (p < 0.05) and a shorter overall survival (OS, p < 0.05). In an *in vitro* study, the regulation of GRP78 in the PDAC cell lines affected the proliferation, migration, and invasion of PDAC cells through the regulation of CyclinD1, cyclin-dependent kinase (CDK) 4, CDK6, phospho-signal transducer, activator of transcription 3 (p-STAT3), janus kinase 2 (JAK2), ras homolog gene family member A (RhoA), Rho-associated kinase 1 (ROCK1), and sterile alpha motif domain containing protein 4 (Smad4). The present data suggest that GRP78 plays a crucial role in the proliferation, migration, and invasion of pancreatic cancer cells and may be a suitable prognostic marker in PDAC.

Pancreatic ductal adenocarcinoma (PDAC) is one of the most aggressive types of malignant tumors, and approximately 46,420 new cases will be diagnosed and approximately 39,590 patients will die of PDAC in the United States in 2014^1^. Although surgery offers the only opportunity for a cure, the prognosis of patients after resection is still disappointing in many cases^2^. The average 5-year survival rate of PDAC patients remains at only approximately 6%^3^; therefore, the identification of new and specific factors that can provide a possible prognosis of PDAC is of great importance. Except for conventional clinicopathological factors, such as lymph node status, perineural invasion (PNI), pathological grade, and stage, many molecules, such as mucin-1 (MUC1), mesothelin (MSLN), and mucin-2 (MUC2), were studied as prognostic biomarkers that are involved in tumor initiation and development. However, more research is needed to accumulate additional data on prognostic biomarkers^4^.

Glucose-regulated protein 78 (GRP78), also known as immunoglobulin heavy chain binding protein, is a member of the heat-shock protein 70 family and is localized in the endoplasmic reticulum (ER). GRP78 has been implicated in the folding and transport of proteins, and plays an essential role as a regulator in ER homeostasis, the activation of the ER stress sensors, and the unfolded protein response (UPR)^5^,^6^. Many investigations have indicated that GRP78 plays a critical role in the proliferation, invasion, and metastasis of human cancer cells^7^,^8^,^9^,^10^,^11^,^12^,^13^,^14^,^15^. Some studies indicated a positive correlation between GRP78 and tumor malignancy (epithelial ovarian tumors^7^, diffuse large B cell lymphoma^8^, renal cell carcinoma^9^, endometrial carcinoma^10^, gastric carcinoma^11^, and prostate cancer^12^), while some other studies came to an opposite conclusion (colorectal carcinoma^13^, oral squamous cell carcinoma^14^, and esophageal adenocarcinoma^15^). Therefore, its effect on the outcome of cancer might be tissue-type specific and involved in different signaling pathways. The correlation between GRP78 expression and the clinicopathological characteristics of PDAC and its role in pancreatic cancer have not been reported.

In the present study, the correlations between GRP78 expression and the clinicopathological characteristics and overall survival of patients with PDAC were investigated using tissue microarray (TMA)-based immunohistochemistry. In addition, the effect of GRP78 expression on the proliferation, migration, and invasion of three PDAC cell lines were evaluated *in vitro*. The changes in the associated proteins were detected by western blotting.

## Results

### Expression of GRP78 and its clinicopathological relevance in human PDAC

**Figure 1:**
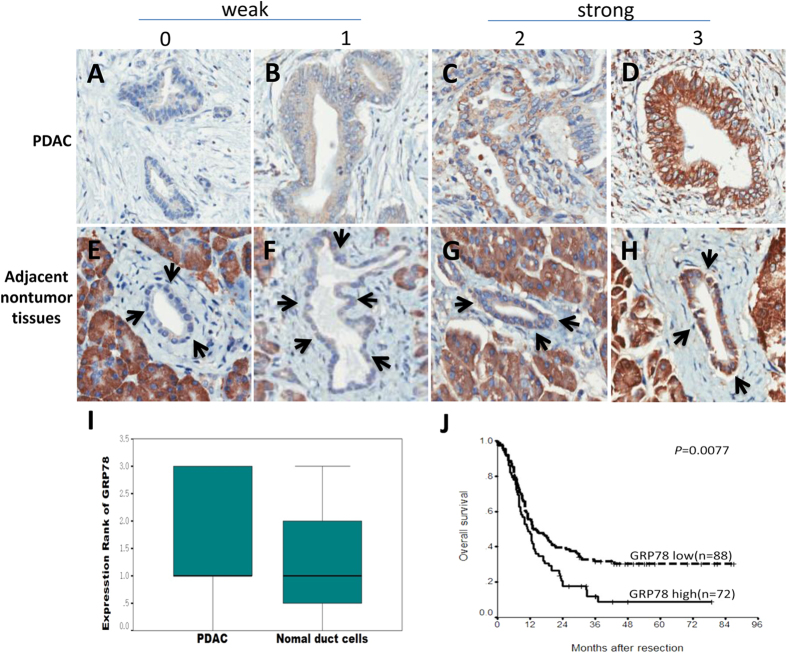
Immunohistochemical staining of GRP78 and the Kaplan-Meier survival analysis. (**A**–**D**) Different staining intensities of GRP78 in the PDAC tumor tissues. (E-H) Different staining intensities of GRP78 in the adjacent nontumor tissues. The cellular staining was classified using a scale of 0-3 as follows: 0 = negative (A, E), 1 = weakly positive (**B**,**F**), 2 = moderately positive (**C**,**G**) and 3 = strongly positive (**D**,**H**) (original magnification 200×). (**I**) Comparison of the GRP78 staining score between the PDAC and nontumor pancreatic duct cells (Mann–Whitney *U*-test, p = 0.009). (**J**) Survival analysis of the PDAC patients after surgical resection based on the GRP78 expression in the tumor tissues (log-rank test, p = 0.0077).

We first examined the expression of GRP78 using tissue microarray-based immunohistochemistry on the tumor tissues and adjacent nontumor tissues from 180 patients with PDAC. GRP78-positive staining was observed in the cytoplasm of both the PDAC cells and in the pancreatic duct cells in the adjacent nontumor tissues. Compared to the normal pancreatic duct cells in the adjacent nontumor tissues, the level of GRP78 staining was significantly higher in the PDAC cells (p < 0.001) (Fig. 1). As shown in Table 1, the expression of GRP78 in the tumor tissue was significantly associated with the tumor (T) stage (p = 0.026), indicating that GRP78 may play a role in the progression of PDAC. No significant association was detected between GRP78 expression and the other clinicopathological characteristics.

### Effect of GRP78 expression on prognosis

**Figure 2:**
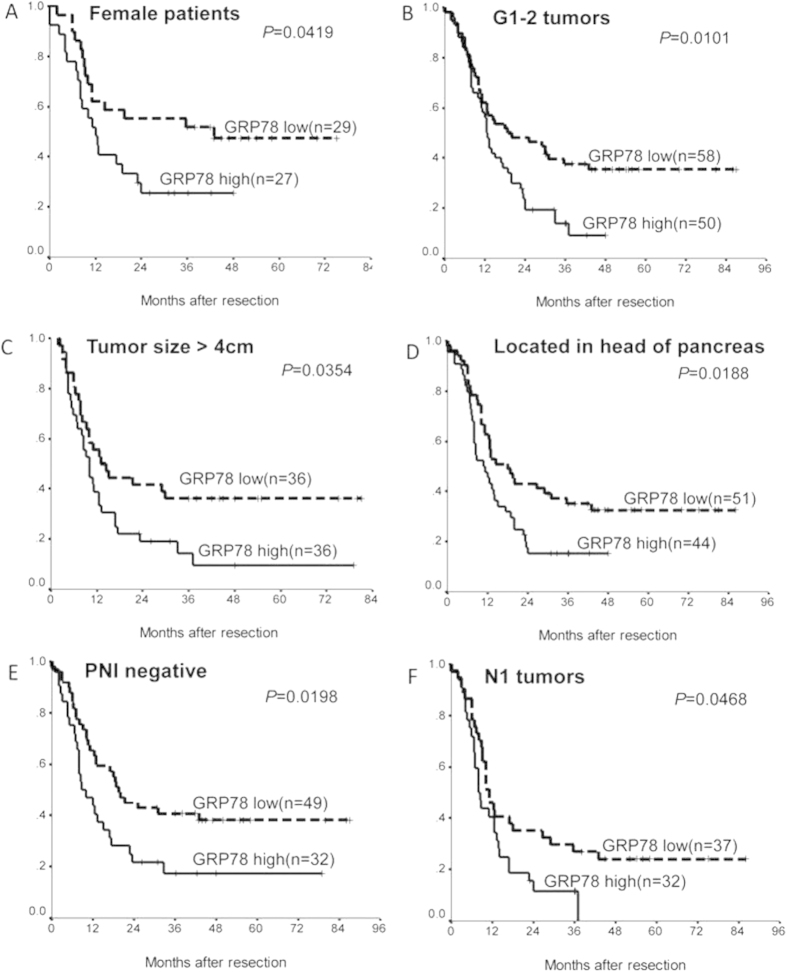
Kaplan-Meier survival analysis of the PDAC patients after surgical resection based on the GRP78 expression in the tumor tissues in many subgroups. (**A**) female patients (p = 0.0419); (**B**) G1-2 tumors (p = 0.0101); (**C**) patients with tumor size > 4 cm (p = 0.0354); (**D**) patients with carcinoma of the head of the pancreas (p = 0.0188); (**E**) PNI-negative tumors (p = 0.0198); (**F**) N1 tumors (p = 0.0468). G1, well differentiated; G2, moderately differentiated; PNI, perineural invasion; N, lymph node.

We next examined the effect of GRP78 expression on prognosis using the Kaplan–Meier method and log-rank test; the univariate analysis showed that the poorer overall survival of patients was significantly associated with high GRP78 expression in the tumor tissues (p = 0.0077) (Fig. 1J), sex (p = 0.0075), histologic grade (p = 0.015), T stage (p = 0.0139), and peripheral nerve infiltration (PNI) (p = 0.0455) (Table 2). The Cox regression analysis showed that GRP78 expression as well as PNI, sex, tumor tissues, histologic grade, and T-stage are independent prognostic markers (p < 0.05) (Table 2). Additionally, the GRP78 expression in the tumor tissues was also an independent prognostic marker (p < 0.05) in many subgroups (female, G1-2, PNI-negative) of PDAC patients (Table 3; Fig. 2).

### Expression of GRP78 in six PDAC cell lines

**Figure 3:**
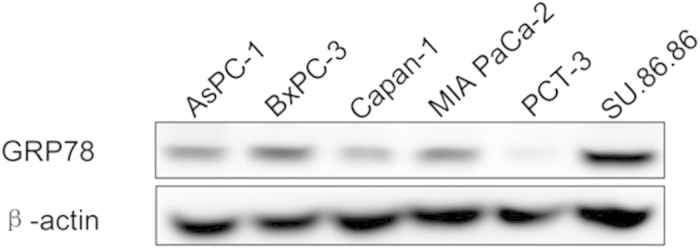
Expression of GRP78 in six PDAC cell lines. Cropped blots/gels were used in the figure and the gels had been run under the same experimental conditions; the full-length blots/gels are presented in Supplementary Figure S3B.

To investigate the effects of GRP78 on the behaviors of PDAC cells, we performed *in vitro* experiments to determine the proliferation and invasion of the cell lines. The expression of GRP78 in six PDAC cell lines, including AsPC-1, BxPC-3, Capan-1, MIA PaCa-2, PCT-3, and SU.86.86 cells, was evaluated by western blotting. As shown in Fig. 3, higher GRP78 expression was observed in the BxPC-3 and SU.86.86 cells. Additionally, relatively low GRP78 expression was observed in the Capan-1 cell line. Therefore, these three cell lines were chosen for additional *in vitro* experiments.

### GRP78 promoted cell proliferation and regulated the cell cycle in PDAC cell lines

**Figure 4:**
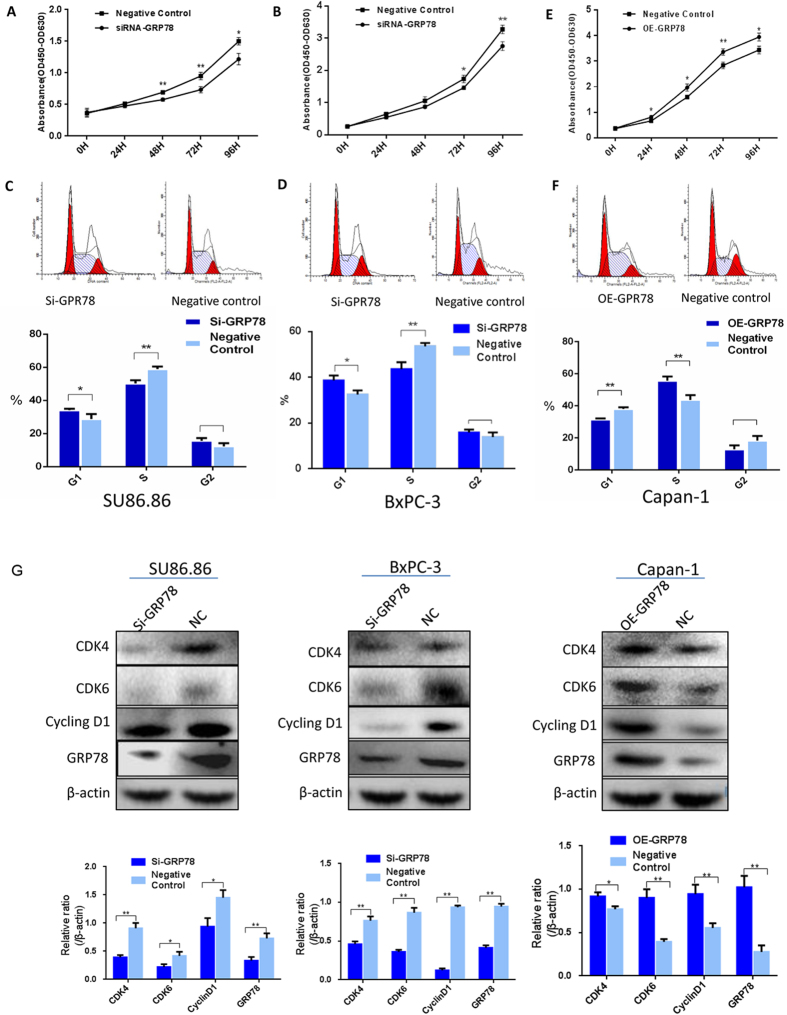
Impact of GRP78 on the proliferation and cell cycle of the PDAC cell lines. (**A**,**B**) The CCK8 assay showed that the proliferation capacity of the SU86.86 and BXPC-3 cells was decreased after GRP78 knockdown. (**C**,**D**) The percentage of SU.86.86 and BXPC-3 cells in G0/G1 phase was significantly increased after GRP78 knockdown. (**E**) The CCK8 assay showed that the proliferation capacity of the Capan-1 cells was increased after forced overexpression of GRP78. (**F**) After the forced overexpression of GRP78, the percentage of Capan-1 cells in G0/G1 phase was significantly decreased, while the percentage of cells in S phase was significantly increased. (**G**) Western blots for CyclinD1, CDK4, and CDK6 in the SU86.86, BXPC-3 and Capan-1 cells after knockdown or overexpression of GRP78 or the negative control. si-GRP78, siRNA-GRP78; OE-GRP78, overexpression of GRP78; NC, negative control. Cropped blots/gels were used in the figure and the gels had been run under the same experimental conditions; the full-length blots/gels are presented in Supplementary Figure S4.

GRP78 was silenced using transient transfection of siRNAs in the BxPC-3 and SU.86.86 cell lines, which highly expressed GRP78. The expression of GRP78 was successfully suppressed at 48 hours after transfection and verified by western blotting (Fig. 4). The CCK8 assay showed that the proliferation of the two GRP78 knockdown cell lines was dramatically reduced compared to the negative control (Fig. 4A,B).

We also evaluated the effect of GRP78 on the cell cycle. Compared to the control group, the downregulation of GRP78 in PDAC cells by transfecting siRNAs significantly decreased the percentage of cells in S phase and increased the percentage of cells in G0/G1 phase (Fig. 4C,D).

Additionally, the Capan-1 cell line was transfected with plasmids to overexpress GRP78 to confirm the effect of GRP78 on cell proliferation. Cell proliferation was significantly increased in the GRP78-overexpressing Capan-1 cell line compared to the negative control (Fig. 4E). Furthermore, the overexpression of GRP78 increased the percentage of cells in S phase in the cell cycle analysis (Fig. 4F).

Western blotting showed that the expression of cell cycle proteins, including CyclinD1, CDK4, and CDK6, was simultaneously suppressed in the GRP78 knockdown groups, while the expression levels of these proteins was upregulated in the GRP78-overexpressing group (Fig. 4G).

### Impact of GRP78 on the invasion and migration of the PDAC cell lines

**Figure 5:**
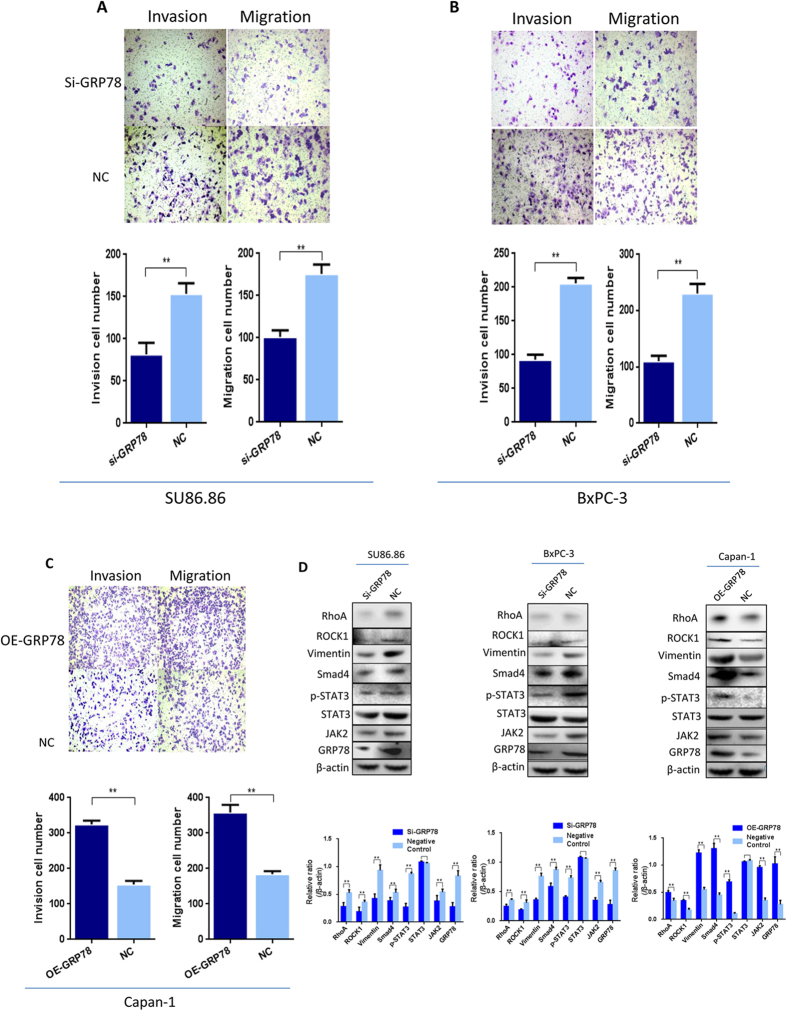
Impact of GRP78 on the invasion and migration of the PDAC cell lines. (**A**) The invasion and migration of the SU.86.86 cells were decreased after GRP78 knockdown compared to the NC group (*p < 0.01). (**B**) The invasion and migration of the BXPC-3 cells were decreased after GRP78 knockdown compared to the NC group. (**C**) The invasion and migration of the Capan-1 cells were increased after the forced overexpression of GRP78 compared to the NC group (*p < 0.01, original magnification, 100×). (**D**) The levels of RhoA, ROCK1, JAK2, vimentin, Smad4, and p-STAT3 were decreased by GRP78 knockdown and increased by GRP78 overexpression in the SU86.86, BXPC-3 and Capan-1 cell lines (*p < 0.05). siRNA, siRNA-GRP78; OE-GRP78, overexpression of GRP78; NC, negative control. Cropped blots/gels were used in the figure and the gels had been run under the same experimental conditions; the full-length blots/gels are presented in Supplementary Figure S5.

After silencing GRP78, the invasion and migration of the BXPC-3 and SU.86.86 cells were significantly reduced compared to those of the negative control by the transwell array (Fig. 5A,B). However, after GRP78 overexpression, the invasion and migration of Capan-1 cells were significantly increased compared to the control group (Fig. 5C).

To examine the mechanisms by which GRP78 regulates cell invasion and migration, we tested the expression of p-STAT3, JAK2, vimentin, RhoA, ROCK1 and Smad4. In the GRP78 knockdown group, the expression of these proteins was downregulated. In contrast, in the GRP78-overexpressing group, the levels of these proteins were significantly increased (Fig. 5D).

## Discussion

Due to the extremely aggressive progression of PDAC, it is important to select prognostic markers that help clinicians to select therapy options that will avoid unnecessary toxicity. GRP78 is involved in cancer development and progression, with tumor-specific characteristics^7^,^8^,^9^,^10^,^11^,^12^,^13^,^14^,^15^. Although it remains unclear how GRP78 participates in tumor proliferation, invasion, and metastasis, a number of studies have reported correlations between GRP78 expression and the clinicopathological characteristics of various human cancers, and the possible mechanisms were explored. Some researchers assume that because of the rapid and unrestrained proliferation of cancer cells, the protein load might exceed the ER capacity, causing the accumulation of misfolded and unfolded proteins in the ER. This causes ER stress and triggers the UPR to restore ER homeostasis by activating a cascade of signaling molecules to induce the ER molecular chaperones and enzymes that enhance the protein folding capacity, and to initiate a process to export and degrade the misfolded and unfolded proteins^16^. As the most well-studied molecular chaperone in the UPR, GRP78 plays an important role in maintaining ER homeostasis and contributes to cancer cell survival and progression. The prognostic significance of GRP78 in human cancers was controversial, according to previous reports^11^,^13^. Increased overall 5-year survival was associated with high GRP78 expression in colorectal cancer^13^, whereas the opposite conclusion was found in gastric cancer^11^ and hepatocellular carcinoma^17^.

A recent study has shown increased GRP78 expression in human PDAC tissue samples^18^. Similar to our results, they also found high expression of GRP78 in normal pancreatic acinar cells^18^, which might be because the acinar cells around the PDAC always suffered from chronic inflammation^19^, and GRP78 has long been found to be highly expressed in inflammation^20^. However, the correlations between GRP78 expression and the clinicopathological characteristics or prognosis of PDAC have not been investigated. In the present study, we tested GRP78 expression in PDAC by immunohistochemistry. Because neoadjuvant chemotherapy might induce ER stress, we excluded those samples. The data showed that compared to the normal pancreatic duct cells, GRP78 was expressed at significantly higher levels in the cytoplasm of PDAC cells, indicating that the cancer cells were often under ER stress and the UPR was triggered to restore ER homeostasis. Moreover, increased GRP78 expression was significantly correlated with a higher tumor (T) stage, suggesting that GRP78 played a crucial role in cancer progression and may serve as a prognostic marker for PDAC patients after surgery. Further analysis revealed that elevated GRP78 expression is associated with a poor prognosis in PDAC patients after surgery, in addition to some conventional clinicopathologic factors, including sex, PNI, histological grade, and T stage. Multivariate analysis proved that GRP78 expression was an independent prognostic factor of overall survival in PDAC. The data from a univariate analysis in some subgroups also supported this conclusion.

We performed *in vitro* experiments to verify our findings. Our findings indicated that GRP78 promoted the proliferation of PDAC cells via elevated expression of CyclinD1, CDK4, and CDK6, which was in accord with a previous report on a renal cell carcinoma cell line^21^. Unrestrained proliferative capacity is a hallmark of cancer^22^. CyclinD1, CDK4, and CDK6 are key proteins that control the G1 checkpoint in cell proliferation^23^,^24^. Our tests on the cell cycle after regulating GRP78 expression reached the same conclusion, suggesting that GRP78 might affect the proliferation of PDAC cells by changing the cell cycle. Overexpression of CyclinD1 and CDK4/CDK6 was also observed in many human malignancies, including gall bladder cancer, endometrial carcinoma, and ovarian serous carcinomas^25^,^26^,^27^,^28^. Moreover, the apoptosis assay was also performed by FCS, but no significant difference was observed, likely because the spontaneous apoptosis of PDAC cell lines occurred at a relatively low level (supplementary material).

The present *in vitro* study also revealed that GRP78 may promote PDAC migration and invasion through a few pathways. The expression of vimentin, a widely used maker for the epithelial-mesenchymal transition (EMT), a common characteristic of cancer cells that contributes to tumor invasion and metastasis^29^, was decreased in accord with the knockdown of GRP78. Vimentin has been reported to be an important prognostic factor for PDAC and to be associated with portal vein invasion and lymph node metastasis^29^. The expression of Smad4 was decreased after the knockdown of GRP78. Smad4 is known as the common mediator of transforming growth factor-β (TGF-β) superfamily signaling, which plays a central role in the EMT. Although Smad4 is generally considered to be a tumor suppressor gene, it is indispensable in the regulation of the TGF-β-inducible EMT. Ya’an, K indicated that the migration and invasion of human pancreatic ductal epithelial cells was diminished by the decreased N-cadherin expression caused by Smad4 knockdown^30^. We found that the expression of RhoA and ROCK1 were synchronously downregulated after GRP78 knockdown. RhoA is a small GTPase protein that is believed to regulate cell motility. Because of its significant role in regulating cell shape, polarity, and locomotion, RhoA and ROCK1 have been well studied in many cancers and proven to promote cancer cell migration and invasion to other tissues^31^. Tavares, AL indicated that TGF-β-mediated RhoA expression is necessary for the EMT^32^. We assume that after knockdown of GRP78, the EMT of the PDAC cells was decreased, causing the decreased invasion and migration capacity, which needs to be investigated in further studies. STAT3 activation has been found to enhance the expression of the vimentin gene^33^, and a close relationship between STAT3 signaling and RhoA has also been found^34^. A study of breast cancer has revealed that the EMT promotes cancer progression via a fibronectin-dependent STAT3 signaling pathway^35^. In our study, the expression of JAK2 and p-STAT3 was downregulated after GRP78 knockdown of GRP78, and upregulated after GRP78 overexpression in PDAC cell lines. The JAK2/STAT3 pathway had been proven to play a crucial role in mediating cancer cell migration and invasion in many human cancers through different mechanisms^36^,^37^,^38^. Guo, K and Li, H found that STAT3 promoted perineural invasion, and STAT3 knockdown could reduce PDAC cell invasion and matrix metalloproteinase-7 expression^39^,^40^.

In summary, the present study showed that the level of GRP78 was elevated in PDAC cells and was associated with a higher T stage and a poor prognosis. In the *in vitro* study, we explored the various mechanisms by which GRP78 might contribute to the proliferation, migration, and invasion of PDAC cell lines. In addition, several reports implicated GRP78 as a potential target in cancer therapy, and corresponding inhibitors have been studied^41^,^42^,^43^,^44^. Our study indicated that GRP78 played an important role in PDAC growth and metastasis, and that it could be a practical prognostic marker and potential target in PDAC therapy.

## Materials and Methods

### Patient information and follow-up

The PDAC tumor tissues and corresponding adjacent nontumor tissues were collected from 180 patients (113 males and 67 females) using the following inclusion criteria: (1) the PDAC samples were histologically proven to be ductal adenocarcinoma by hematoxylin–eosin staining; (2) both the paired tumor and nontumor tissues were obtained; and (3) the patients were not undergoing neoadjuvant chemotherapy. The diagnosis and staging were based on the Staging Manual of the American Joint Committee on Cancer, 7^th^ edition. The adjacent nontumor tissue was excised from 3 cm around the cancerous tissue. The exclusion criteria included patients with other organic diseases or the inability to provide informed consent. Of the 180 patients, follow-up data was obtained for 160 cases (approximately 0–87 months; median: 12.55 months), as shown in Tables 1 and 2. Overall survival was defined as the survival time after surgery. This study was approved by the Ethics Committee of Peking Union Medical College Hospital and adhered to the tenets of the Declaration of Helsinki. Written informed consents for the storage of the tissue samples and publication of this study were obtained before surgery after a complete verbal explanation.

### Tissue microarray construction and immunohistochemistry

The TMA was constructed with a manual tissue microarray (Beecher Instruments, Sun Prairie, WI, USA) using formalin-fixed paraffin-embedded blocks. Two cores of the tumor and adjacent nontumor tissue for each case were sampled from representative areas using a 1.5-mm punch.

Immunohistochemistry was performed to detect GRP78 expression. A rabbit GRP78 monoclonal antibody (ab108613, Abcam Biotech Company, Cambridge, UK) and an EnVision+ two-step staining kit (Dako, Glostrup, Denmark) were used for staining. Briefly, the 4-μm-thick formalin-fixed, paraffin-embedded tumor sections were mounted, deparaffinized in xylene, and rehydrated in a graded alcohol series. The slides were washed with phosphate-buffered saline (PBS), immersed in a 0.01 mol/L citrate buffer solution (pH 6.0), and heated in a microwave for 10 min for antigen retrieval. Peroxidase was quenched using 3% hydrogen peroxide for 10 min. After washing again with PBS, the slides were incubated at 4 °C overnight with the primary antibody (1:50). After washing with PBS four times, the horseradish peroxidase-conjugated secondary antibody was added for 30 min and diaminobenzidine was used as a chromogen. Finally, the slides were counterstained with hematoxylin (Sigma-Aldrich Co., LLC, Munich, Germany) to visualize the nuclei. Preimmune rabbit serum was used at the same dilution as the negative control.

### Staining evaluation

A blinded staining evaluation was performed by two experienced pathologists who did not have access to the clinicopathological or follow-up data. Based on a previous study^13^, the frequencies of GRP78 staining were almost at the same level in the tissues; the staining intensity was used to evaluate the expression of GRP78. The proportion of stained cells was first classified using a scale of 0–3 as follows: 0 = negative, 1 = weakly positive, 2 = moderately positive and 3 = strongly positive. Finally, GRP78 expression was simply summarized as low (intensity 0 or 1) or high (intensity 2 or 3) as shown in Fig. 1.

### Cell lines and culture conditions

The human PDAC cell lines AsPC-1, BxPC-3, Capan-1, MIA PaCa-2, PCT-3, and SU.86.86 were donated by the University of Heidelberg. The cells were cultured in HyClone RPMI-1640 medium (GE Healthcare Life Sciences, Logan, Utah, USA) supplemented with 10% HyClone fetal bovine serum (FBS, GE Healthcare Life Sciences, Logan, Utah, USA) in a humidified incubator with 5% CO_2_ at 37 °C.

### Small interfering RNA and transfection

The small interfering RNA for GRP78 (5′-CUAUGAAGCCCGUCCAGAAtt-3′) was purchased from Thermo Fisher Scientific Corporation (Waltham, MA, USA), and a nonsense sequence that is unrelated to siGRP78, 5′-UUCUCCGAACGUGUCACGUtt-3′, was used as a negative control. Lipofectamine 2000 (Thermo Fisher Scientific, Waltham, MA, USA) was used for transfection according to the manufacturer’s instructions. The SU.86.86 and BxPC-3 cells were plated in 6-well plates at a concentration of 5 × 10^5^ cells/well in 2 mL of medium supplemented with 10% FBS. After 18 h, when the confluence of cells reached approximately 80%, the cells were used for transient transfection. A mixture containing the 80 pM of the siGRP78 duplexes and the Lipofectamine 2000 reagent was added to each well for transfection. The cells were collected after 48 h.

### Plasmids and transfection

The recombinant vectors encoding human GRP78 were constructed by PCR-based amplification and were then subcloned into the pcDNA3.1 expression vector (Thermo Fisher Scientific, Waltham, MA, USA). The vectors were transfected into the cells using Lipofectamine 2000 (Thermo Fisher Scientific, Waltham, MA, USA) according to the manufacturers’ protocol.

### Western blot analysis

To obtain the whole-cell lysates, the transfected cells were digested with a 0.25% trypsin solution and lysed with RIPA buffer (Thermo Fisher Scientific, Waltham, MA, USA) containing a protease and phosphatase inhibitor cocktail (Sigma-Aldrich Co., LLC, St. Louis, MO, USA). The protein concentrations were measured with a BCA protein assay kit (Thermo Fisher Scientific, Waltham, MA, USA). The total proteins (50 μg) were first separated on 10% SDS-PAGE and then electrotransferred to a polyvinylidene difluoride membrane (PVDF, Millipore Corporation, Billerica, MA, USA). The PVDF membranes were blocked with 5% nonfat dry milk in TBS/Tween 0.5% (TBS-T) for 1 h, and then incubated overnight with primary antibodies at 4 °C. After washing with TBS-T three times for 5 min, the membranes were incubated in horseradish peroxidase–conjugated mouse or rabbit secondary antibodies (1:3,000; Golden Bridge Biological Technology, Beijing, China) in 5% (w/v) nonfat dry milk in TBST for 1 h at room temperature. The protein bands were detected using an enhanced chemiluminescence system (Merck & Co., Inc. Whitehouse Station, NJ, USA) and the intensities were quantified using Quantity One software. Equal protein sample loading was guaranteed by incubating the membranes with a β-actin antibody as the internal control standard. The anti-human-GRP78 primary antibody was used at a dilution of 1:1000 as described above. The anti-human-CyclinD1, anti-human-CDK4, anti-human-CDK6, anti-human-p-STAT3, anti-human-STAT3, anti-human-Smad4, anti-human-JAK2, anti-human-vimentin, anti-human-RhoA, and anti-human-ROCK1 primary antibodies were purchased from Cell Signaling Technology (Danvers, MA, USA) and used at a dilution of 1:500.

### Cell proliferation analyses

Cell proliferation was detected using the CCK-8 kit (Dojindo Laboratories, Kumamoto, Japan). Briefly, the cells were seeded in flat-bottomed 96-well plates at 1000 cells/well with 100 μL medium, 10 μL of the CCK-8 solution was added to each well, and the plates were incubated for 3 h at 37 °C. The absorbance was measured at 450 nm using the SpectraMax 190 microplate reader (Molecular Devices, LLC, Sunnyvale, CA, USA) at time points from days 1 to 4. A wavelength of 630 nm was used as a reference.

### Cell cycle assays

The cell cycle analysis was performed by fluorescence-activated cell sorting. The PDAC cells were harvested at 24 h after transfection. After washing with cold phosphate-buffered saline (PBS), the cells were resuspended in ice-cold PBS, and fixed in 70% ice-cold ethanol overnight at 4 °C. The cells were stained with a solution containing 0.2 mg/ml RNase A, 0.1% Triton X-100 and 20 μg/ml propidium iodide and analyzed using the Accuri C6 flow cytometer (Becton Dickinson, New Jersey USA).

### Migration and invasion assays

The cell migration and invasion assays were performed using a double-chamber assay with a pore size of 8 μm (Corning Life Sciences, New York, NY, USA). The membranes for the invasion assay were coated with diluted Engelbreth-Holm-Swarm murine sarcoma extracellular matrix proteins (1:8, E1270, Sigma-Aldrich Co., LLC, St. Louis, MO, USA) and then incubated at 37 °C for 4 h. According to the manufacturer’s protocol, the cells (4 × 10^4^ cells per well) were added to the upper chambers containing serum-free media, and 600 μL of medium containing 10% FBS was added to the lower chamber. The cells for migration assays were carefully removed from the upper side of the membrane with a swab after incubating for 12 hours, and the cells for the invasion assays were removed after incubating for 24 hours. The migration and invasion activity was assessed by counting the number of cells on the lower side of the filter. The membranes were fixed with methanol and then stained with crystal violet. The number of cells in five randomly selected fields was counted under a light microscope at a magnification of 200×. All *in vitro* experiments were independently repeated in triplicate under identical conditions.

### Statistical analyses

A McNemar’s test and the Mann–Whitney *U*-test were used to compare GRP78 staining between the tumor tissues and the adjacent nontumor tissues. A chi-square test was used to detect the correlations of the categorical variables. The Kaplan–Meier method and log-rank test were used to evaluate the survival analysis, and the Cox regression model was used to analyze the prognostic factors. The Student’s *t*-test was used to determine the significance of the differences in the *in vitro* cell invasion, migration, cell cycle and apoptosis assays. SPSS (Statistical Package for the Social Sciences version 11.05; SPSS Inc., Chicago, IL, USA) was used for the statistical analyses. P < 0.05 was defined as being statistically significant.

## Additional Information

**How to cite this article**: Niu, Z. *et al.* Elevated GRP78 expression is associated with poor prognosis in patients with pancreatic cancer. *Sci. Rep.* **5**, 16067; doi: 10.1038/srep16067 (2015).

## Supplementary Material

Supplementary Information

## Figures and Tables

**Table 1:** Association between the GRP78 expression in the tumor tissues and the clinicopathologic variables of PDAC.

Variable		GRP78 expression	p value*
	No. of cases	Low high	
Age (years)			0.361
≥65	62	37 25	
<65	118	62 56	
Gender			0.566
Male	113	64 49	
Female	67	35 32	
Tumor location			0.876
Head	101	54 47	
Body/tail	67	35 32	
Tumor size			0.079
>4 cm	76	35 41	
≤4 cm	94	56 38	
Histological grade			0.406
G1–2	120	62 58	
G3	37	22 15	
pT stage			**0.026**
T1–2	96	58 38	
T3–4	74	32 42	
pN stage			0.487
N0	95	52 43	
N1	71	35 36	
Perineural invasion			0.111
Positive	84	40 44	
Negative	87	52 35	

Note: The boldface type indicates a significant difference. PDAC, pancreatic ductal adenocarcinoma; G1, well differentiated; G2, moderately differentiated; G3, poorly differentiated; T, tumor; N, lymph node. * Chi-square test.

**Table 2:** Univariate and multivariate Cox regression analyses of the association of the prognosis with the clinicopathological parameters and GRP78 expression in PDAC.

Variable	No. of cases	Univariate analysis			Multivariate analysis		
		Median ± SE	95% CI	p value#	RR	95% CI	p value*
Age (years)				0.9272			
≥65	53	11 ± 2	7–15				
<65	107	13 ± 1	10–15				
Gender				**0.0075**			**0.041**
Male	104	11 ± 1	9–13		1.617	1.017–2.187	
Female	56	14 ± 5	5–24		1		
Tumor location				0.4050			
	95	13 ± 1	10–16				
	60	11 ± 1	8–14				
Tumor size				0.9543			
>4 cm	94	13 ± 1	10–16				
≤4 cm	69	11 ± 1	8–14				
Histological grade				**0.0150**			**0.013**
G1–2	108	14 ± 2	9–19		1		
G3	36	**9 ± 1**	7–11		1.696	1.116–2.576	
pT stage				**0.0139**			**0.036**
T1–2	94	13 ± 4	6–20		1		
T3–4	64	11 ± 2	8–14		1.508	1.026–2.514	
pN stage				0.0778			
N0	91	15 ± 3	10–20				
N1	62	10 ± 2	7–13				
Perineural invasion				**0.0455**			0.348
Positive	77	11 ± 1	9–13		1.204	0.817–1.776	
Negative	81	15 ± 3	9–21		1		
GRP78 in tumor				**0.0077**			**0.041**
High	72	11 ± 1	8–14		1.491	1.017–2.187	
Low	88	13 ± 4	6–20		1		

Note: The boldface type indicates a significant difference. PDAC, pancreatic ductal adenocarcinoma; SE, standard error; RR, relative risk; CI, confidence interval; G1, well differentiated; G2, moderately differentiated; G3, poorly differentiated; T, tumor; N, lymph node. ^#^Log-rank test; *Cox regression test.

**Table 3:** Multivariate Cox regression analyses of the prognostic relevance of GRP78 expression in PDAC subgroups in which the poorer overall survival of patients was significantly associated with high GRP78 expression (identified by the log-rank test).

Subgroups	RR	95% CI	p value *
Females	2.222	1.031–4.790	**0.042**
G1–2	1.808	1.136–2.876	**0.012**
Size > 4 cm	1.668	0.824–3.378	0.155
Head of pancreas	1.639	0.996–2.697	0.052
PNI negative	2.101	1.143–3.863	**0.017**
N1	1.763	0.955–3.254	0.070

Note: The boldface type indicates a significant difference. PDAC, pancreatic ductal adenocarcinoma; RR, relative risk; CI, confidence interval; G1, well differentiated; G2, moderately differentiated; PNI, perineural invasion; N, lymph node. *Cox regression test.
