# Supplementary material for: Elevated GRP78 expression is associated with poor prognosis in patients with pancreatic cancer
Source: Sci Rep. 2015 Nov 4;5:16067. doi: 10.1038/srep16067 (PMC4632002; doi:10.1038/srep16067)
Supplement: Supplementary Information [file srep16067-s1.pdf]

Elevated GRP78 expression is associated with poor prognosis in patients with pancreatic cancer

Zheyu Niu, Mengyi Wang, Li Zhou, Lutian Yao, Quan Liao*, Yupei Zhao*

Supplemental results

The specificity of anti-GRP78 antibody was verified by using blocking peptide

Anti-GRP78 antibody was blocked after incubating with blocking peptide at room temperature for 30 minutes. Compared with positive control, no stain of anti-GRP78 antibody was observed in IHC of the PDAC tissues (Fig.S1A-D). The Western blot analysis of the cell lines demonstrated less non-specific bands was found using ab108613 than ab168806. After incubating with blocking peptide of ab108613 for 30 minutes, the Western blot analysis of the cell lines demonstrated absence of GRP78 bands. (Fig.S1E).

No significant difference was observed in apoptosis analyses of PDAC cell lines

The results showed that knockdown or forced overexpression of GRP78 did not influence apoptosis and death of PDAC cells without induction of UV rays or chemicals (Fig.S2). After knockdown of GRP78, the apoptosis rate of SU86.86 was (8.73±0.26)%, while the apoptosis rate of control group was (7.77±0.32)%, no significant difference was observed (p = 0.078). Similar results were observed in BxPC-3 group (p = 0.096) and forced overexpression group (Capan-1, p = 0.539).

Verification of knockdown of GRP78 by three different siRNAs by western blotting.

The knockdown of GRP78 was performed by using three different siRNAs. The sequences of the siRNAs were: siRNA-1, 5'-CUAUGAAGCCCGUCCAGAAtt-3'; siRNA-2, 5'-GAAUGAAUUGGAAAGCUAUTT-3'; siRNA-3, 5'-GGAGCGCAUUGAUACUAGATT-3'; Negative control, 5′-UUCUCCGAACGUGUCACGUtt-3′. Western blotting verified that siRNA-1 was the most effective one which was chosen for the further experiments (Fig S3).

Supplemental Material and Methods

Verification for specificity of anti-GRP78 antibody. Blocking peptide (ab176143, Abcam Biotech Company, Cambridge, UK) for anti-GRP78 antibody (ab108613, Abcam Biotech Company, Cambridge, UK) was used according to the manufacturers' protocol. For Western blotting of the whole protein of SU86.86 cell line, antibody and blocking peptide were added to dilution buffer at a dilution of 1:1000, incubate at room temperature for 30 minutes before reacting with the blot. For immunohistochemistry of PDAC tissues, twice the volume of peptide as volume of antibody was used. Positive control was performed without blocking peptide.

Apoptosis analyses. Apoptosis analyses of PDAC cells were assessed by flow cytometric analysis of cells stained with Annexin-V-FITC and propidium iodide (PI) according to the manufacturer's instruction (BD Biosciences) using the Accuri C6 flow cytometer (Becton Dickinson, New Jersey USA) after transfected for 24h.

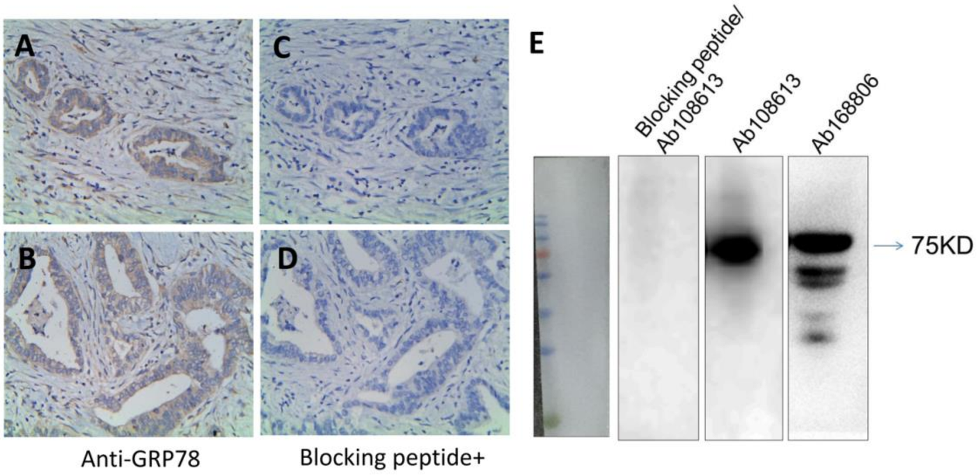

Figure S1. The specificity test of anti-GRP78 antibody by immunohistochemistry and western blotting using blocking peptide. (A-D) After incubating with blocking peptide for 30 minutes, no stain of anti-GRP78 antibody was observed in IHC of the PDAC tissues compared with positive control. (E) Less non-specific bands was found using ab108613 than ab168806. After incubating with blocking peptide of ab108613 for 30 minutes, the Western blot analysis of the cell lines demonstrated absence of GRP78 bands.

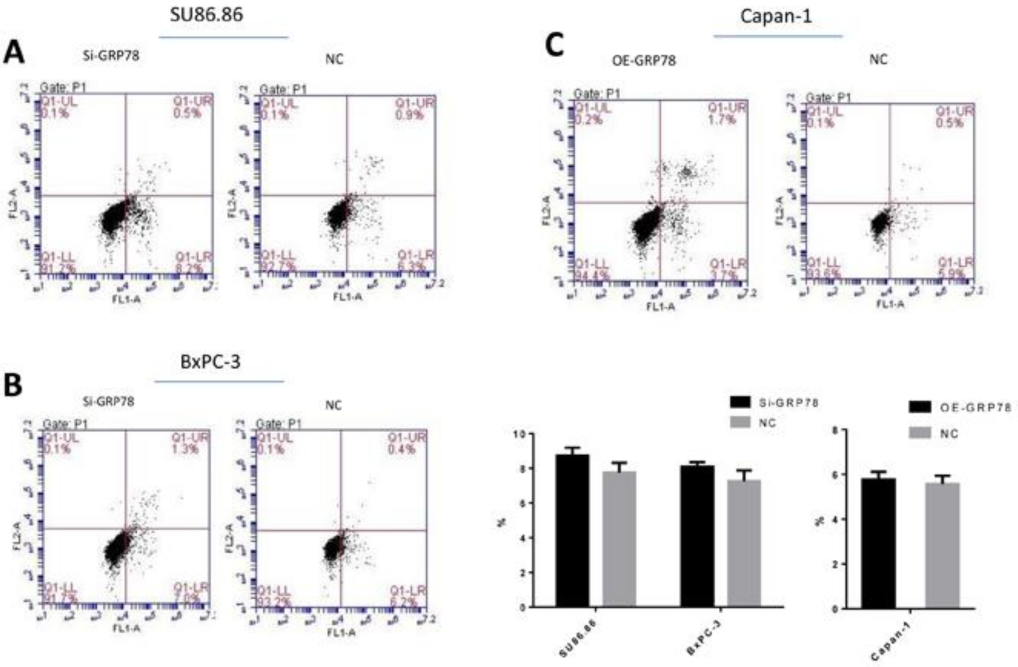

Figure S2. Impact of GRP78 on apoptosis of PDAC cell lines. (A,B) No significant difference was observed in apoptosis analyses of SU.86.86 and BXPC-3 cells after knockdown of GRP78 compared with NC group (SU.86.86, p = 0.078; BXPC-3, p = 0.096); (C) No significant difference was observed in apoptosis analyses of Capan-1 cells after overexpression of GRP78 compared with NC group (p = 0.539).

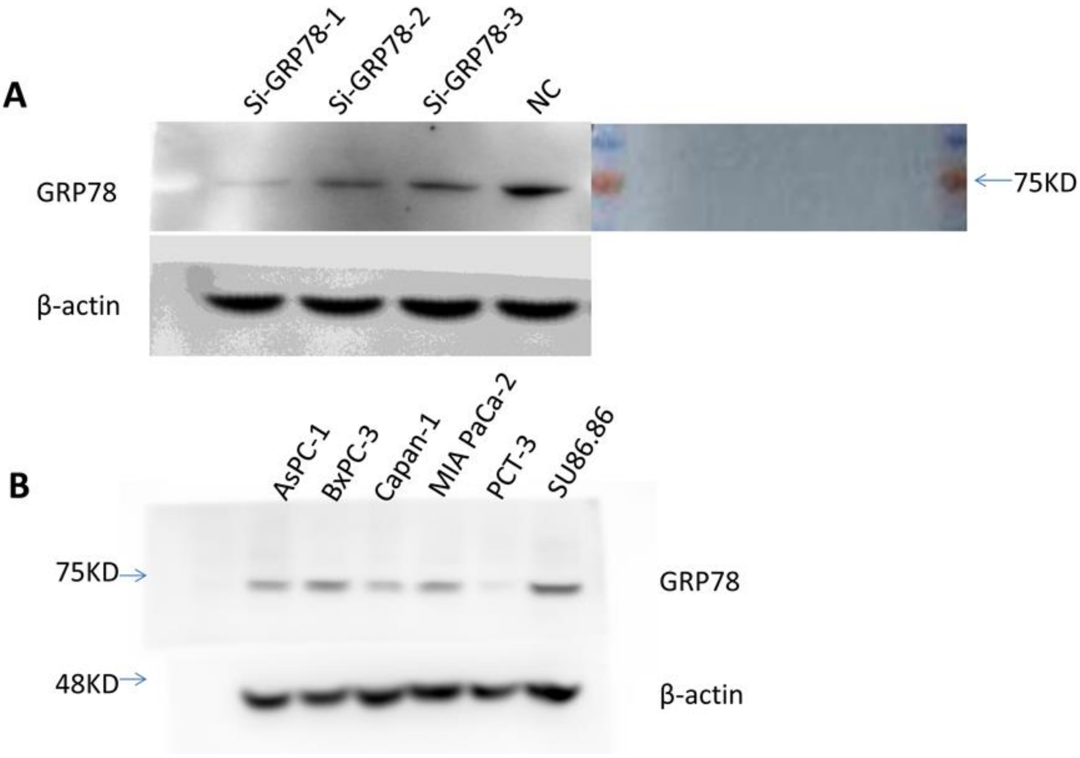

Figure S3. Verification of knockdown of GRP78 by three different siRNAs by western blotting.

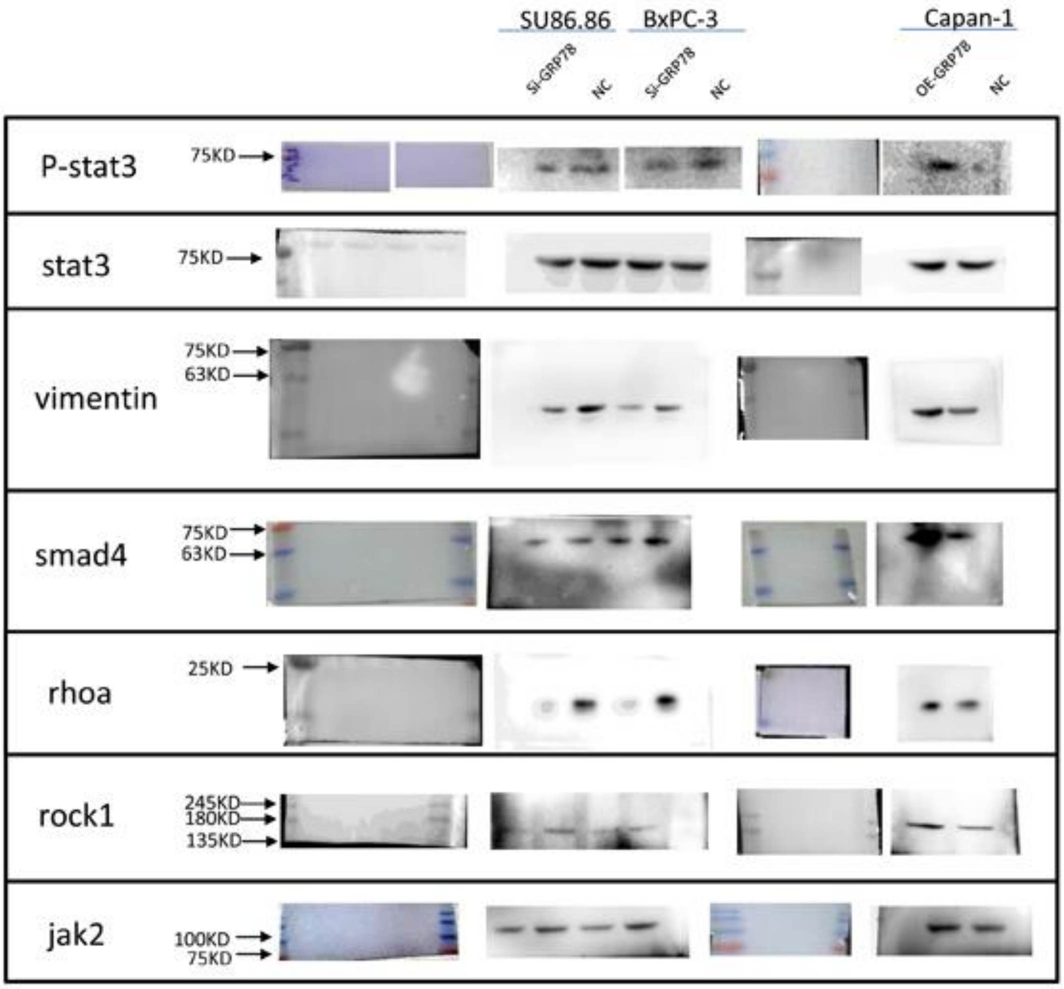

Figure S4. Original western blotting of figure 4G.

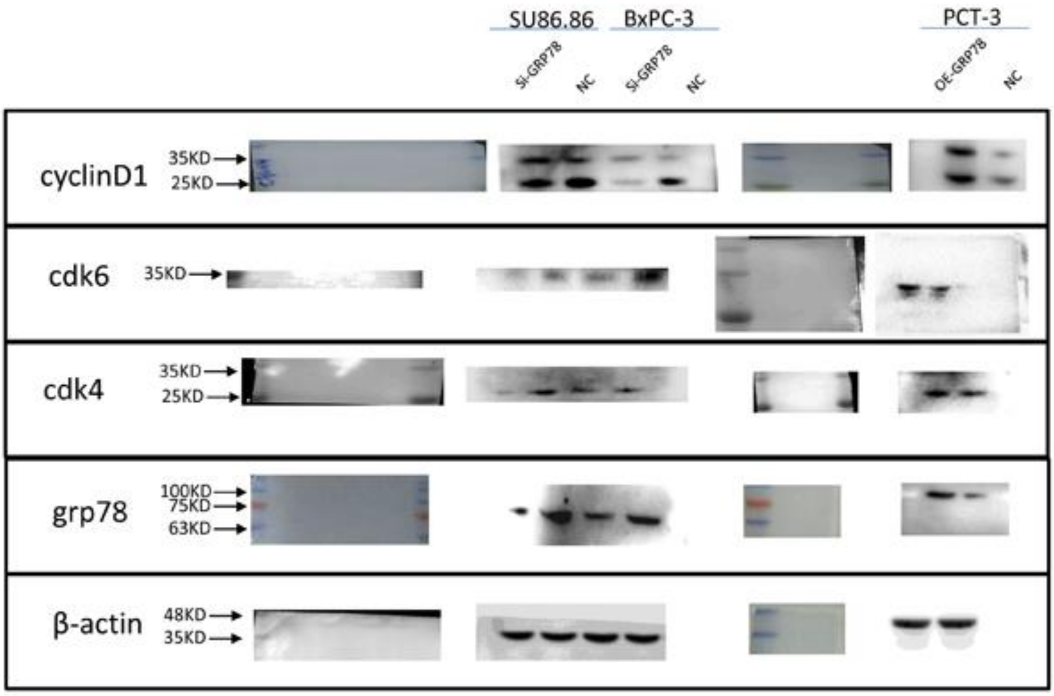

Figure S5. Original western blotting of figure 5D.
